# Stem cell-based treatments against stroke: observations from human proof-of-concept studies and considerations regarding clinical applicability

**DOI:** 10.3389/fncel.2014.00357

**Published:** 2014-10-29

**Authors:** Thorsten R. Doeppner, Dirk M. Hermann

**Affiliations:** Department of Neurology, University of Duisburg-Essen Medical SchoolEssen, Germany

**Keywords:** stem cells, stroke, mesenchymal stem cells, neural progenitor cells, trials

## Abstract

Ischemic stroke remains a heavy burden for industrialized countries. The only causal therapy is the recanalization of occluded vessels via thrombolysis, which due to a narrow time window still can be offered only to a minority of patients. Since the majority of patients continues to exhibit neurological deficits even following successful thrombolysis, restorative therapies are urgently needed that promote brain remodeling and repair once stroke injury has occurred. Due to their unique properties of action, stem cell-based strategies gained increasing interest during recent years. Using various stroke models in both rodents and primates, the transplantation of stem cells, namely of bone marrow derived mesenchymal stem cells (MSCs) or neural progenitor cells (NPCs), has been shown to promote neurological recovery most likely via indirect bystander actions. In view of promising observations, clinical proof-of-concept studies are currently under way, in which effects of stem and precursor cells are evaluated in human stroke patients. In this review we summarize already published studies, which due to the broad experience in other medical contexts mostly employed bone marrow-derived MSCs by means of intravenous transplantation. With the overall number of clinical trials limited in number, only a fraction of these studies used non-treated control groups, and only single studies were adequately blinded. Despite these limitations, first promising results justify the need for more elaborate clinical trials in order to make stem cell transplantation a success for stroke treatment in the future.

## Stem and progenitor cells in experimental stroke models

In defined areas of the adult brain such as the subgranular zone of the dentate gyrus and the subventricular zone (SVZ) of the lateral ventricles, endogenous neurogenesis persists during adulthood (Alvarez-Buylla and Garcia-Verdugo, 2002; Taupin and Gage, 2002; Silva-Vargas et al., 2013; Braun and Jessberger, 2014; Jessberger and Gage, 2014; Sawada et al., 2014). These neurogenic niches comprise astrocyte like neural stem and progenitor cells (NPC) that give rise to neurons under physiologic conditions (Doetsch et al., 1997, 1999). In rodent and primate stroke models, cerebral ischemia stimulates endogenous neurogenesis, and NPCs migrate from the SVZ towards the site of injury where they proliferate and differentiate into neurons (Liu et al., 1998; Arvidsson et al., 2002; Tonchev et al., 2003; Yamashita et al., 2006). Unfortunately, both differentiation and survival rates of endogenous NPCs are low (Parent, 2003; Haas et al., 2005; Doeppner et al., 2009). For this reason, the functional contribution of endogenous neurogenesis to post-ischemic neurological recovery remains a matter of debate.

In view of their restorative properties, efforts have been made to promote neurological recovery by transplantation of stem or progenitor cells in ischemic stroke. Although embryonic stem cells have the capacity to give rise to all cell lineages, their therapeutic potential is limited due to teratoma formation and ethical concerns (Blum and Benvenisty, 2008, 2009). Consequently, a wide variety of adult stem and progenitor cells from different species and various tissue sources have been used for therapeutic purposes, which were transplanted either locally or systemically in experimental models of focal cerebral ischemia (Bacigaluppi et al., 2008, 2009; Schwarting et al., 2008; Bliss et al., 2010; Zheng et al., 2010; Banerjee et al., 2012; Doeppner et al., 2012; Leong et al., 2012). Although grafted cells are not thought to be integrated into residing neural networks, they do promote neurological recovery via paracrine (indirect) mechanisms that involve the stimulation of endogenous angioneurogenesis and neural plasticity, stabilization of the blood brain barrier as well as modulation of peripheral and central immune responses (Doeppner et al., 2012; Hermann and Chopp, 2012; Mora-Lee et al., 2012; Zhang and Chopp, 2013). Despite the fact that questions related to the optimal cell type, the most adequate cell delivery time point and the route of cell delivery are still vividly discussed, clinical trials have been on the way with some of them holding promising results. Due to the broad experience with these cells in other clinical contexts, the most widely used cell source in clinical stroke studies are mesenchymal stem cells (MSCs), which are mostly derived from bone marrow. Besides, NPCs are also used in stroke patients. The following paragraphs provide an overview on major clinical trials (Table 1), which due to the existence of multiple investigator-driven smaller trials by no means claims to be exhaustive.

**Table 1:** **Clinical trials using stem cells or progenitor cells against stroke**.

Authors	Year	Cell type	Key findings
Bang et al.	2005	MSCs	Cells were intravenously grafted twice within 9 weeks post-stroke. Better outcome in Barthel index after 1 year, but no effect on NIHSS and MRI scan.
Lee et al.	2010	MSCs	Intravenous cell grafting twice post-stroke with observation period of 5 years. Better outcome in mRS.
Bhasin et al.	2011	MSCs	Autologous intravenous MSC transplantation. Within 24 weeks, no significant side effects observed plus putative increased neural plasticity.
Bhasin et al.	2013	MSCs	Intravenous MSC transplantation followed by observation period of 24 weeks. Statistically improved modified Barthel Index and increased neural plasticity after stem cell treatment. No side effects.
Honmou et al.	2011	MSCs	Intravenous cell transplantation showed no side effects during 1 year of follow-up. Reduction of lesion volume by >20% after 1 week.
Savitz et al.	2011	MSCs	Intravenous transplantation of MSCs within 72 h post-stroke plus observation period of 6 months. No study-related side effects. Median NIHSS 13 before cell grafting and 3 after 6 months.
Savitz et al.	2010	MSCs	Intraarterial delivery of 99mTc-labeled MSCs. Significantly reduced intracerebral numbers of grafted cells after 24 h. No significant side effects for as long as 120 days.
Moniche et al.	2012	MSCs	Intraarterial infusion of MSCs between 5-9 days post-stroke. After 6 months, no side effects but also no improved functional outcome.
Suárez-Monteagudo et al.	2009	MSCs	Stereotactic transplantation of cells into 5 patients. Authors claim discrete functional improvement after 1 year.
Kondziolka et al.	2000	Cultured neuronal cells	Stereotactic delivery of cells with observation period of 18 months. Some functional improvement. No relevant safety issues.
Kondziolka et al.	2005	Cultured neuronal cells	Stereotactic cell delivery with maximal observation period of 24 months. Some functional improvement, but primary outcome was not met. No significant adverse events.
Savitz et al.	2005	Fetal lateral eminescence (=neural) cells	Cells were pre-treated with anti-MHC I antibody and intracerebrally delivered. Study was stopped after 5 patients. Significant side effects.
Rabinovich et al.	2005	Cell suspension from immature nervous and hemopoietic tissue	Intrathecal cell delivery in 10 patients. No significant side effects during 6 months of observation.

*The table describes the studies quoted in the main text with special regard to key findings, the cell type used and the year of publication. MRI: magnetic resonance imaging, mRS: modified Rankin Scale, MSCs: mesenchymal stem cells, NIHSS: National Institutes of Health Stroke Scale*.

## Clinical stroke trials using stem or progenitor cells

### Mesenchymal stem cell sources

MSCs have been successfully used under various experimental paradigms (Chen et al., 2001, 2003; Li et al., 2002; Kurozumi et al., 2005; Ukai et al., 2007; Onda et al., 2008; Yoo et al., 2008; Kranz et al., 2010; Sheikh et al., 2011). Due to the long-lasting experience with MSC transplantation in other clinical contexts, namely in malignancies of the blood, MSCs have evolved as the preferred candidate for clinical transplantation studies. Characteristics of MSCs include adherence on plastic surfaces, expression of CD markers such as CD105, CD73 and CD90 as well as differentiation into fat, bone and cartilage tissue (Dominici et al., 2006). MSCs can be easily obtained from various tissue sources including bone marrow and adipose tissue (Bliss et al., 2007; Doeppner and Hermann, 2010). They can also be easily expanded *in vitro* and are regarded to be immunologically inert, which reduces the risk of rejection of grafted cells in allogeneic transplantation settings (Aggarwal and Pittenger, 2005; Beyth et al., 2005). Although MSCs can be induced to differentiate into neural tissue *in vitro* (Pittenger et al., 1999), their potential for neural differentiation is low. Nevertheless, transplantation of MSCs improves neurological outcome in experimental stroke models, which is attributed to paracrine effects of grafted cells (Caplan, 2009). As such, promising pre-clinical data lead to clinical studies using MSCs or bone marrow-derived mononuclear progenitor cells (subsequently subsumed together as MSCs), the most relevant of which are presented in the next paragraph.

### Clinical proof-of-concept studies using mesenchymal stem cell transplantation

In one of the very first randomized controlled phase I/II clinical trials, Bang et al. (2005) intravenously transplanted autologous MSCs twice within 9 weeks after stroke onset. During the observation period of 1 year, patients receiving MSCs showed better improvement of the Barthel Index than control patients, whereas National Institutes of Health Stroke Scale (NIHSS) score and brain injury assessed by magnetic resonance imaging (MRI) did not differ between groups. Patient numbers in this trial were low (5 MSC transplanted and 25 control patients), thus possibly explaining the lack of significant results in the latter readouts. Taking this lack of statistical power and concerns regarding the safety of fetal calf serum (Spees et al., 2004) that was used for *ex vivo* MSC expansion into account, the same study group performed an additional open-label and observer-blinded clinical trial on patients suffering from severe strokes using an observation period of 5 years (Lee et al., 2010). As in their earlier study, MSCs were intravenously transplanted twice in a total of 16 patients, whereas controls received no injection. Although 4 patients from the MSC group died during the observation period, no significant side effects or comorbidities attributed to the transplantation itself were observed. Importantly, the modified Rankin Scale (mRS) score was significantly improved in patients receiving MSCs within the observation period. This improvement was associated with increased levels of stromal cell-derived factor-1 that is upregulated upon stroke in rodents and thought to be involved in MSC homing (Shen et al., 2007). Although Lee et al. (2010) significantly extended the observation period to 5 years, more definite conclusions about the efficacy of MSCs cannot be drawn from this study, which lacked randomization and true blinding. Interestingly however, beneficial effects related to MSC treatment seemed to correlate with the involvement of the SVZ into the stroke. Thus, MSC transplantation was more effective when the SVZ was not part of the evolving stroke lesion, suggesting an indirect action of MSCs that promote endogenous neuroregeneration. According to a recent MRI study, the SVZ is located in close proximity to stroke lesions in a large percentage of stroke patients. In a prospective cohort of 108 included patients with first-ever stroke, the distance from the nearest margin of the infarct to the SVZ was ≤2 mm in half of all patients exhibiting visible diffusion weighted image (DWI) lesions (Delavaran et al., 2013). Thus, the relationship between stroke lesion and SVZ may represent a hitherto under-recognized factor influencing responses to neurorestorative therapies (Lee et al., 2010).

In the meantime, further clinical trials provided evidence that intravenous MSC transplantation is safe and feasible in humans (Bhasin et al., 2011, 2013; Honmou et al., 2011; Savitz et al., 2011). However, scientific conclusions from these trials are hampered due to the heterogeneous study design, the different timing of cell delivery and considerable differences in size of study groups. As a matter of fact, only two of the aforementioned studies included non-treated control groups (Bhasin et al., 2011; Savitz et al., 2011). Nevertheless, these studies were not randomized or blinded, and beneficial effects due to MSC transplantation as described by Bhasin et al. therefore need further evaluation.

Since the homing of MSCs and other stem cells into the brain is limited after intravenous transplantation, a Brazilian group performed intraarterial infusions of ^99m^Tc-labeled MSCs in six stroke patients with the aim of increasing the amount of grafted cells within the brain (Barbosa da Fonseca et al., 2010). Although significant cell homing was observed as early as 2 h post-stroke within the ischemic hemisphere, the latter was greatly diminished 24 h post-stroke. Within an observation period of 120 days, no significant side effects related to cell grafting were observed. Similar findings were reported by Battistella et al. (2011) for an observation period of 180 days after intraarterial MSC infusion during the chronic phase of the stroke. Noteworthy, conclusions from the aforementioned studies are limited by low patient numbers and a lack of appropriate blinding. Since MSCs are known to home into peripheral organs such as the lungs, questions remain about the safety of required interventional procedures, particularly in more severely affected patients suffering from serious comorbidities. Patient safety will have to be taken into account carefully in future treatment studies after a recent study did not observe a beneficial effect of intraarterial MSC transplantation in patients suffering from stroke (Moniche et al., 2012). In animal studies, intraarterial MSC delivery was not superior to intravenous delivery (Yang et al., 2013).

In an even smaller clinical study, Suarez-Monteagudo et al. successfully transplanted autologous MSCs stereotactically into the brain of five stroke patients, followed by an observation period of 1 year (Suárez-Monteagudo et al., 2009). Although the authors claimed a discrete functional improvement over time, a scientific evaluation of this observation is certainly misplaced due to the low patient number and the study trial itself with no adequate control group. A sample of five patients is even far too low to draw conclusions about therapeutic safety. Reports of neurological improvement in small patient cohorts are hampered by the fact that complication rates may not be adequately determined. The MSC delivery into the brain parenchyma should, if at all, be considered with great caution.

At present, further studies that analyze both safety and feasibility of MSCs in stroke patients are on the way. The U.S. National Institutes of Health^1^ currently list 10 clinical trials ranging from phases I to III upon the keywords “mesenchymal stem cells” and “stroke”. The majority of these studies use intravenous cell delivery with primary outcome measurements of either safety or functional neurological improvement. However, the recruitment status of these studies remains heterogeneous with some trials having not yet recruited at all.

### Critical considerations regarding mesenchymal stem cell transplantation in humans

Although clinical follow-up studies with observation periods of one or 5 years did not show significant side effects (Bang et al., 2005; Lee et al., 2010), safety issues carefully need to be taken into account when considering MSCs for the treatment of ischemic stroke. MSC treatment was associated with improved outcome of stroke patients in at least some of the aforementioned studies, suggesting that MSC transplantation is safe. Likewise, a large meta-analysis on clinical trials under various pathological conditions not exclusively related to stroke did not show any evidence for severe side effects due to MSC transplantation (Lalu et al., 2012). This work analyzed safety issues in 36 studies covering a total of 1012 participants that had suffered from stroke, Crohn’s disease, cardiomyopathy, myocardial infarction or graft vs. host disease. Some studies also included MSC transplantation in healthy non-affected volunteers. The meta-analysis did not detect significant side effects related to MSC transplantation, such as acute infusion-related toxicity, complications in peripheral organ systems, infection, death, or tumor formation. There was, however, a consistent observation of MSC transplantation-related transient fever.

Nevertheless, setbacks and unfavorable reports in experimental stroke models deserve special attention. Using intraarterial transplantation paradigms in a rat stroke model, Mitkari et al. could not show improved functional outcome after MSC transplantation, although grafted MSCs were attracted towards the lesion site and post-ischemic angiogenesis was significantly increased (Mitkari et al., 2014). In line with this, Steiner and colleagues did not observe post-stroke neuroprotection after systemic MSC transplantation in rodents, which was attributed to homing of grafted MSCs into peripheral organs and not into the brain (Steiner et al., 2012). Most importantly, transplantation risks might increase when comorbidities such as diabetes are taken into account. As such, Chen et al. did not show a beneficial effect of MSCs in diabetic stroke rats, but even reported increased mortality in treated animals that was associated with enhanced brain hemorrhage as a consequence of maladaptive angiogenesis (Chen et al., 2011).

Although MSCs themselves are not tumorigenic, they might migrate to existing primary tumors and modify or even stimulate tumor growth due to their immunomodulatory properties (Lazennec and Jorgensen, 2008). Accordingly, MSC-induced bystander effects might change the biological behavior of tumor cells with unpredictable consequences for the patient. Hence, further clinical trials using larger study cohorts with extended observation periods are urgently needed before more definite conclusions about the safety of MSC transplantation may be drawn. In view of risks related to invasive procedures, such studies should preferably use intravenous instead of delivery strategies from the authors’ point of view.

### Neural stem and progenitor cells and other stem cell sources

In comparison with MSCs, other cell types have been less frequently evaluated in clinical studies. The hesitation of delivering these cells to human stroke patients is a consequence of the fact that unlike MSCs, which are used for bone marrow transplantation, these cells had not been used before as therapeutics in other medical contexts. In case of NPCs, concerns remain about malignant transformation, which cannot be ruled out completely even when fetal or adult cell sources are used. NPCs derived from the SVZ of the lateral ventricles induce potent neuroprotection and brain remodeling, both when systemically and locally (i.e., intracerebrally) delivered. This aspect deserves special attention, since cells are not integrated into neural networks but act mainly via paracrine bystander mechanisms (Bacigaluppi et al., 2008, 2009; Doeppner et al., 2010, 2012). Two clinical trials have demonstrated the feasibility of stereotactic delivery of cultured neuronal cells derived from a teratocarcinoma cell line (Kondziolka et al., 2000, 2005). Although cell transplantation was followed by some functional improvement, the primary outcome measure of the study, the change in the European Stroke Scale (ESS) motor score, was not met. Again, the sample size (4–7 patients per group) was too small to infer conclusions regarding the therapeutic efficacy of NPC transplantation.

Another study using fetal porcine NPCs pre-treated with an anti-MHC class I antibody for prevention of graft rejection could not confirm its high expectations regarding safety and feasibility (Savitz et al., 2005). Noteworthy, this trial was stopped after intracerebral transplantation into five patients resulted in significant side effects in two patients. Thus, temporary worsening of motor deficits was noted in one patient 3 weeks after transplantation, while another patient developed epileptic seizures 1 week after transplantation (Savitz et al., 2005). MRI in both patients demonstrated areas of contrast enhancement remote from the grafting site, which resolved on subsequent imaging. In contrast to this study, another study investigating the intrathecal transplantation of cell suspensions derived from immature nervous and hematopoietic tissues did not detect any side effects in 10 patients over an observation period of 6 months (Rabinovich et al., 2005). Although the number of published clinical studies investigating cell sources other than MSCs is low, additional studies are on the way in stroke patients using cells from different tissue sources. Among these ones, studies using genetically modified NPCs are noteworthy, which are delivered by stereotactic intracerebral transplantation (NCT02117635 and NCT01151124). In other studies, human placenta-derived cells (NCT01310114) or olfactory ensheathing cells (NCT01327768) are applied. While the former study is already completed but not yet published, the status of the latter is currently unknown.^2^ Unfortunately, the aforementioned studies make use of intraparenchymal transplantation strategies, which reduces their clinical relevance, even in case they prove to be successful.

## Conclusion and outlook

Whereas application of stem cells has become a clinical routine for treatment of hematological diseases, neurorestaurative treatment paradigms using stem cells against stroke have not found their way into the clinic, yet. However, first proof-of-concept studies evaluating MSC transplantation in human stroke patients achieved promising data, which justify more systematic studies. These observations will have to be confirmed in larger cohorts in the near future, before more definite conclusions regarding the safety of stem cell treatment can be made. Unfortunately, recruitment in some of the ongoing studies was rather slow in recent years, which delays progress in the field of stem cell therapies. A major problem of several previous studies is the lack of appropriate control groups. On the other hand, clinical trials using cell sources other than MSCs are still scarce and need further evaluation. In the absence of clinical experience in larger patient cohorts, questions of long-term safety remain a concern for most of the latter cell sources, even when fetal or adult cells are used.

## Author and contributions

Thorsten R. Doeppner and Dirk M. Hermann wrote the manuscript.

## Conflict of interest statement

The authors declare that the research was conducted in the absence of any commercial or financial relationships that could be construed as a potential conflict of interest.

## Footnotes

^1^www.clinicaltrials.gov

^2^www.clinicaltrials.gov
